# Risk Factors of Immune-Mediated Hepatotoxicity Induced by Immune Checkpoint Inhibitors in Cancer Patients: A Systematic Review and Meta-Analysis

**DOI:** 10.3390/curroncol31110525

**Published:** 2024-11-13

**Authors:** Ying Jiang, Ranyi Li, Xiaoyu Li, Ningping Zhang

**Affiliations:** 1 Department of Pharmacy, Zhongshan Hospital Fudan University, Shanghai 200032, China; jiang.ying5@zs-hospital.sh.cn (Y.J.); li.ranyi@zs-hospital.sh.cn (R.L.); 2 Department of Gastroenterology, Zhongshan Hospital Fudan University, Shanghai 200032, China

**Keywords:** immunotherapy, immune-mediated hepatotoxicity, incidence, risk factor, meta-analysis

## Abstract

Immune checkpoint inhibitors (ICIs) significantly improve survival, while immune-mediated hepatotoxicity (IMH) has been reported. To evaluate the incidence and potential risk factors of IMH among cancer patients treated by ICIs, PubMed/Medline, Web of Science, Cochrane, and Embase were searched before 30 March 2024 for systematic review and meta-analysis. Odds ratios (ORs) with 95% confidence intervals (CI) were calculated. Quality assessment was completed using the Newcastle–Ottawa scale. Of 1217 articles identified, 24 consisting of 9076 patients were included, with one study being prospective and the rest retrospective. The overall incidence of any grade IMH and grade ≥ 3 secondary to ICIs was 14% and 7%, respectively. The cholestatic pattern was more prevalent than the hepatocellular and mixed patterns. The meta-analysis revealed that ICI treatment was related to reduced risk of IMH in older patients (SMD: −0.18; 95% CI: −0.33 to −0.04), individuals with higher body mass index (WMD: −2.15; 95% CI: −3.92 to −0.38), males (OR: 0.44; 95% CI: 0.27 to 0.72), and patients with lung cancer (OR: 0.58, 95%CI 0.41 to 0.83). On the other hand, patients with liver metastasis (OR: 1.80; 95% CI: 1.47 to 2.20), history of ICI treatment (OR: 3.09; 95% CI: 1.21 to 7.89), diabetes (OR: 2.19; 95% CI: 1.36 to 3.51), chronic HBV (OR: 3.06; 95% CI: 1.11 to 8.46), and concomitant use of ICIs (OR: 8.73; 95% CI: 2.41 to 31.59) increased the risk of developing IMH. This study will provide clinicians with information on potentially high-risk groups for IMH, who need to be cautiously monitored for liver function when receiving immunotherapy.

## 1. Introduction

Immune checkpoint inhibitors (ICIs) are monoclonal antibodies that possess anticancer properties by blocking the binding of intrinsic regulators on the surface of T cells. Specifically, they target the anti-programmed death receptor 1 (PD-1) and the cytotoxic T-lymphocyte-associated antigen 4 (CTLA-4), preventing their interaction with their ligands, PD-L1/PD-L2 and CD80/CD86, respectively. This blockade restores T-cell activation and revitalizes T-cell-mediated tumor cell surveillance and destruction [1]. ICIs have emerged as the major anticancer regimens in addition to surgery, chemotherapy, radiotherapy, and small-molecule-targeted therapy and considerably improved the survival rate and prognosis in patients with refractory tumors, including melanoma (MM), non-small-cell lung cancer (NSCLC), renal cell carcinoma (RCC), and hepatocellular carcinoma (HCC). The field of immunotherapy is evolving, and recent research indicates that the anti-tumor effectiveness of ICIs is significantly augmented by innovative antibody–drug conjugates (ADCs). A fundamental study indicates that ADC drugs, in conjunction with immunotherapy, can enhance PD-L1 expression and perhaps mitigate resistance to either agent, thus providing a novel paradigm in oncological treatment [2,3,4,5].

Despite durable anti-tumor effects, ICIs occasionally cause a range of unintended adverse events (AEs) that are termed immune-related adverse events (irAEs), resulting from the excessive immune response targeting normal tissues or organs, which undermine the efficacy of ICI therapy [6]. Among individual ICIs, the most common irAEs affect the cutaneous, endocrine, cardiovascular, and gastrointestinal systems, including the liver [7]. Immune-mediated hepatotoxicity (IMH) induced by ICIs is generally asymptomatic, with an incidence rate ranging from 1% to 15% in randomized trials [8,9]. Typically, it is identified incidentally through routine liver function tests during post-treatment monitoring. Moreover, IMH has emerged as a significant concern within drug-induced liver injury (DILI) since it was considered a distinct and specific subtype of DILI [10]. Ongoing research continues to reveal additional clinical features related to IMH. A deeper understanding of IMH development and its clinical presentation can guide evidence-based therapy strategies for clinicians.

Despite the low severity of most IMH cases, life-threatening cases related to acute liver failure and even death have been reported during phase III clinical trials and post-marketing surveillance [11,12,13]. The overall estimated mortality rate for irAE ranges from 0.3% to 1.3% [7]. Data from the VigiLyze–VigiBase database of the World Health Organization indicate that 20.2% (124/613) of patients who experienced fatal ICI-related toxic events died from IMH, underscoring the urgent need for early detection and effective management of these events [14]. Numerous studies have explored the risk factors of IMH in patients receiving ICIs, and a comprehensive treatment approach has been proposed based on these findings. However, substantial disagreements remain regarding the risk factors that occur after ICI treatment. This inconsistency arises from differences in data collected from clinical trials and real-world cohorts and variations in the included population. Therefore, this systematic review and meta-analysis aimed to determine the incidence, prevalence, and risk factors of IMH following immunotherapy to provide treatment options for cancer patients at potential risk.

## 2. Methods

This work followed the PRISMA statement and has been registered in the PROSPERO (CRD42024531146).

### 2.1. Search Strategy and Data Extraction

Relevant articles that investigated the risk factors of IMH in patients receiving ICIs were comprehensively searched in PubMed, Embase, Web of Science, and Cochrane Library before 30 March 2024 by two independent authors (Y. J and RY. L) using Medical Subject Heading (MeSH) terms (“Immunotherapy”, “Risk factors”, “Chemical and Drug Induced Liver Injury”) and corresponding free words, with additional customized fields such as “pembrolizumab”, “Keytruda”, “MK-3475”, and “tislelizumab”. Supplementary Appendix S1 for risk factors provides detailed search strategies for each database. There were no limitations on study design and publication language.

### 2.2. Inclusion and Exclusion Criteria

Articles meeting the following criteria were included: (a) included individuals diagnosed with malignancy who received treatment with ICIs, either alone or in combination; (b) provided information on the number of patients with any grade of IMH and those who did not develop IMH; (c) examined the risk factors associated with IMH; and (d) the full text was available. The publications were excluded based on the following criteria: (a) duplication of research, guidelines, consensus, letters, comments, notes, reports, case reports, conference abstracts, reviews, meta-analyses, experimental studies, and studies that were irrelevant or had insufficient data and (b) studies that did not have the full text available.

### 2.3. Data Extraction and Quality Assessment

Two researchers (Y. J and RY. L) independently reviewed the titles, abstracts, and full texts to screen for eligible articles. For any discrepancies in study selection, discussions and consultations were conducted with the third researcher (NP. Z). The following information was extracted from the included articles: (a) basic details such as first author, publication year, region, data source, study design, sample size, criteria for evaluating AEs, tools for assessing the causality between ICIs and IMH, and IMH definition; (b) adjusted odds ratios (ORs) and 95% confidence intervals (CI) of potential risk factors, encompassing age, gender combination therapies of anti-PD-(L)1 and anti-CTLA-4, comorbidities, liver metastasis, liver cirrhosis, and HCC; and (c) additional characteristics of both groups were further extracted due to the limited studies providing the adjusted ORs, including demographic features, cases in each group, number of IMH patients for all grades, ICI types, presence of liver metastasis, history of ICIs treatment, underlying liver disease, treatment regime (monotherapy or combined therapy), performance status score, and laboratory tests. Data were documented using a standardized extraction sheet.

The article quality was appraised with the Newcastle–Ottawa scale (NOS) by three authors (Y. J, RY. L, and NP. Z) through 8 questions comprising selection (0–4 scores), comparability (0–2 scores), and outcome (0–3 scores) aspects. A NOS score of 1–3, 4–6, and 7–9 indicated low, moderate, and high quality, respectively.

### 2.4. Statistical Analysis

All calculations were performed and all graphs were created using Stata Statistical Software version 17.0. The reported sample size and IMH events were utilized to calculate incidence, and the inverse variance method was applied to pool IMH incidence. Subgroup analyses were conducted to determine the influence of region, data source, cancer types, ICI types, and the criteria of IMH. The risk factors for any grade of IMH were also investigated. Extracted continuous data, expressed as median with range or interquartile, were converted to mean with standard deviation for pooling. Heterogeneity across studies was assessed using the Cochrane Q test and inconsistency index (*I*^2^) statistics. An *I*^2^ statistic < 50% or a *p* value > 0.05 implied low heterogeneity, warranting the use of fixed-effect, while a higher I^2^ statistic necessitated the adoption of the random effects model. Egger’s tests were utilized to evaluate publication bias, with *p* < 0.05 indicating notable publication bias. Additionally, sensitivity analyses were performed. All analyses were considered statistically significant at *p* < 0.05.

## 3. Results

### 3.1. Study Selection and Characteristics

**Figure 1:**
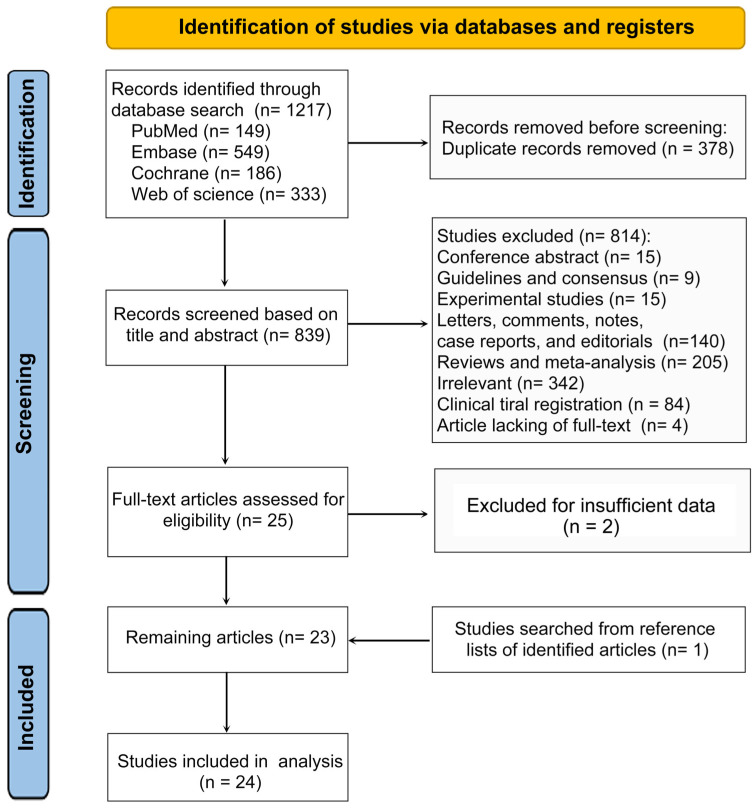
Flowchart of the selection process followed PRISMA guidelines.

A total of 1217 articles were retrieved after a comprehensive search conducted up to 31 March 2024, including 149 records from PubMed, 549 from Embase, 186 from Cochrane, and 333 from Web of Science (Figure 1). After removing 378 duplicates, 839 studies were retained for the initial title and abstract screening. Among these, 814 articles were excluded for conference abstract (*n* = 15); guidelines and consensus (*n* = 9); experimental studies (*n* = 15); letters, comments, notes, case reports, and editorials (*n* = 140); reviews and meta-analysis (*n* = 205); irrelevant studies (*n* = 342); clinical trial registration (*n* = 84); inability to access the full text (*n* = 4); and insufficient data (*n* = 2). Furthermore, one study was added by searching for eligible articles from the reference lists. Moreover, Celsa et al. compared the characteristics and outcomes of immunotherapy-related liver injury in patients with HCC and other advanced solid tumors [15]. To enhance the accuracy of patient information extraction, the meta-analysis divided the research, and the characteristics of the populations are presented in Table 1. Finally, 24 studies comprised the meta-analysis, resulting in a pooled cohort of 9076 participants.

The characteristics of the included studies are manifested in Table 1 [1,15,16,17,18,19,20,21,22,23,24,25,26,27,28,29,30,31,32,33,34,35,36,37]. Only one study was conducted prospectively [15], while the rest was retrospective [1,16,17,18,19,20,21,22,23,24,25,26,27,28,29,30,31,32,33,34,35,36,37]. Among the included studies, 23 reported IMH of any grade [1,15,16,17,18,19,20,21,22,23,24,25,26,28,29,30,31,32,33,34,35,36,37], and 15 reported grade ≥ 3 IMH [15,16,17,20,21,23,24,25,27,31,32,33,34,35,37]. This study covered various cancer types, including HCC, MM, RCC, urothelial cancer (UC), head and neck cancer, and lung cancer. Several studies reported immune-related adverse events in addition to IMH. One study reported adverse events in all patients, including gastrointestinal, endocrine, skin, lung, neuromuscular, and rheumatologic events [15]. Nine studies documented concurrent irAEs in patients with IMH, with the most prevalent irAEs being endocrine, gastrointestinal, skin, and colitis (Table S1).

### 3.2. Quality Assessment

Table S2 summarizes the quality of the studies. Of these, 17 were classified as high quality, with scores ranging from 7 to 9. The other studies were categorized as moderate quality due to their deficiencies in providing follow-up information and adequately addressing confounding factors.

### 3.3. Risk Factors for IMH Following ICIs

**Figure 2:**
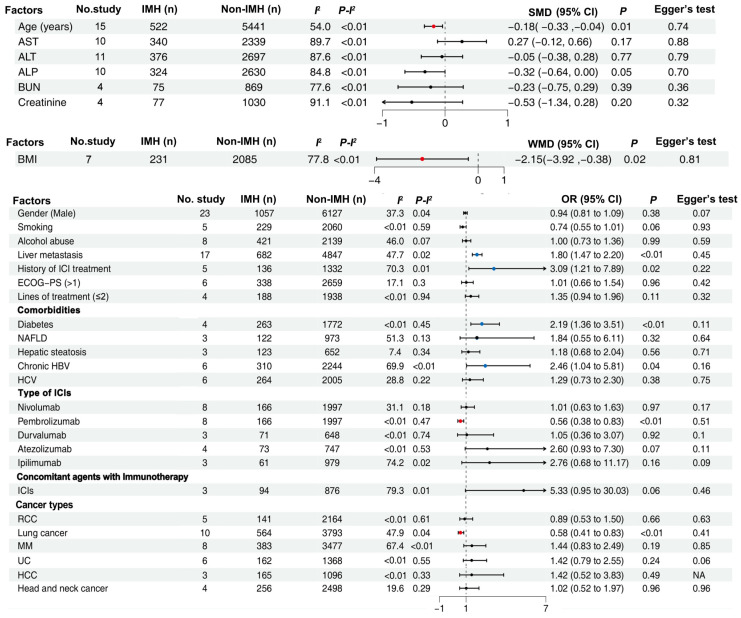
Meta-analysis of risk factors for IMH in patients with malignancies. Red and blue dots indicate the significance of factors. Red dots signify that the factors reduce the risk of IMH, while blue dots indicate that the factors increase the risk of IMH.

**Figure 3:**
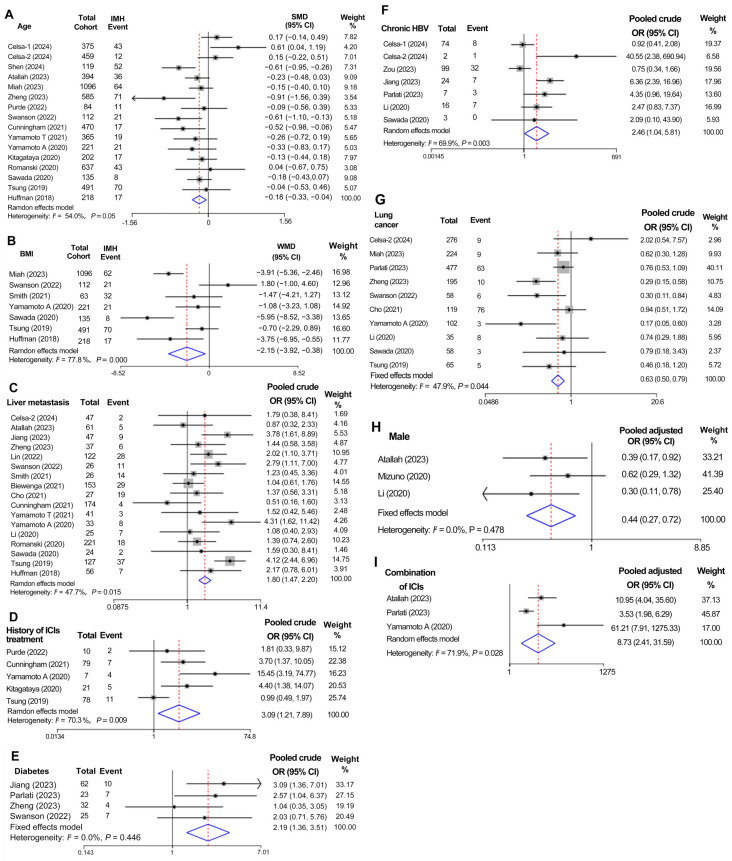
Forest plot of summary results of risk factors for IMH in patients treated with ICIs. (**A**) Age (*n* = 15); (**B**) BMI (*n* = 7); (**C**) patients with liver metastasis (*n* = 17); (**D**) patients with history of ICI treatment (n = 5); (**E**) diabetes (*n* = 4); (**F**) chronic HBV (*n* = 6); (**G**) lung cancer (*n* = 10); (**H**) gender of male (*n* = 3); (**I**) combination of ICIs (*n* = 3). The blue rhombus signifies the pooled effect size.

The baseline characteristics of patients in the IMH and non-IMH groups were compared across 24 studies (Figure 2), and the significant factors were extracted and illustrated in Figure 3 as a forest plot.

#### 3.3.1. Age, BMI, and Gender

As shown in Figure 2, the risk of IMH post-ICI therapy is generally associated with a younger age (SMD: −0.18; 95% CI: −0.33 to −0.04; *I*^2^ = 54.0%; *p* = 0.012; *n* = 15 studies) and lower BMI (WMD: −2.15; 95% CI: −3.92 to −0.38; *I*^2^ = 77.8%; *p* = 0.017; *n* = 7 studies) in patients who received ICI treatment, and the forest plots are presented in Figure 3A and 3B, respectively. No significant publication bias was detected for either factor, according to Egger’s test. Figures S1 and S2 illustrated the sensitivity analyses regarding the sources of heterogeneity for age and BMI. Moreover, male gender was related to a reduced risk of IMH (pooled adjusted OR: 0.44; 95% CI: 0.27 to 0.72; *I*^2^ = 0%; *p* = 0.001; *n* = 3 studies) (Figure 3H and Table S3).

#### 3.3.2. Liver Metastasis

A total of 17 studies reporting liver metastasis were pooled in a meta-analysis, resulting in a pooled OR of 1.80 (95% CI: 1.47 to 2.20; *I*^2^ = 47.7%; *p* < 0.000), with no significant publication bias (*p* = 0.451) (Figure 2 and Figure 3), indicating a significantly elevated risk of IMH in those patients treated with ICIs.

#### 3.3.3. History of ICI Treatment

The number of patients who had received prior ICI treatment was reported in five trials, which showed statistical differences between patients with IMH and those without (pooled OR: 3.09; 95% CI: 1.21 to 7.89). Of note is that substantial heterogeneity was observed across these five studies (*I*^2^ = 70.3%) (Figure 2 and Figure 3D). Sensitivity analyses addressing the source of heterogeneity were presented in Figure S3.

#### 3.3.4. Comorbidities

Within the pooled cohort, the risk of IMH after ICI treatment was notably higher in cancerous individuals with diabetes (pooled OR: 2.19; 95% CI: 1.36 to 3.51; *I*^2^ = 0.0%; *p* = 0.001; *n* = 4 studies) (Figure 3E) and chronic HBV (pooled OR: 2.46; 95% CI: 1.04 to 5.81; *I*^2^ = 69.9%; *p* = 0.039; *n* = 7 studies) (Figure 3F), but not in those with NAFLD (pooled OR: 1.84; 95% CI: 0.55 to 6.11; *I*^2^ = 51.3%; *p* = 0.321; *n* = 3 studies), hepatic steatosis (pooled OR: 1.18; 95% CI: 0.68 to 2.04; *I*^2^ = 7.4%; *p* = 0.562; *n* = 3 studies), and HCV (pooled OR: 1.29; 95% CI: 0.73 to 2.30; *I*^2^ = 28.8%; *p* = 0.384; *n* = 6 studies). Consistent with the results from the pooled binary variable on chronic HBV, chronic HBV (pooled adjusted OR: 3.06; 95% CI: 1.11 to 8.46; *p* = 0.031) was also a significant risk factor for IMH, without marked heterogeneity (*I*^2^ = 41.1%) and publication bias (*p* = 0.370) after pooling the adjusted ORs from four studies (Table S3).

#### 3.3.5. Cancer Types

Immunotherapy with ICIs was used for various types of cancer, such as lung cancer, RCC, UC, MM, head and neck cancer, and HCC. Among them, lung cancer (564/3793) and MM (383/3477) were the most frequently reported. The meta-analysis further investigated the risk factors for IMH, with a specific emphasis on various cancer types. Lung cancer had a reduced risk of IMH development compared to other cancer types (OR: 0.58; 95% CI: 0.41 to 0.83; *I*^2^ = 47.9%; *p* = 0.003; n = 10 studies), without any substantial difference in heterogeneity (Figure 3G).

#### 3.3.6. Concomitant Agents with Immunotherapy

In some patients, immunotherapy was combined with additional medications such as tyrosine kinase inhibitors (TKIs), immune ICIs, proton pump inhibitors (PPIs), and nonsteroidal anti-inflammatory drugs (NSAIDs). The impact of concomitant ICIs was analyzed (Figure 3I). When pooling the adjusted ORs, three studies indicated that the combination of ICIs (pooled adjusted OR: 8.73; 95% CI: 2.41 to 31.59; *I*^2^ = 71.9%; *p* = 0.001), specifically CTLA-4 and PD-(L) inhibitors, was significantly associated with an increased risk of any grade IMH (Table S3).

### 3.4. Incidence of IMH

The mean time to onset of any grade IMH within the pooled cohort was 2.16 months (95% CI: 1.18 to 3.13). The estimated incidence of IMH following ICI treatment was 14% (95% CI: 11% to 17%; *I*^2^ = 94.9%) for any grade and 7% (95% CI: 5% to 8%; *I*^2^ = 91.0%) for grades above 3 (Table 2). Given substantial heterogeneity, subgroup analyses were conducted regarding the region, data source, cancer types, ICI types, and criteria of AEs, and none of them were revealed to be independent contributors (Figure S4). In terms of clinical patterns, the cholestatic pattern (52%; 95% CI: 34% to 70%) tended to be marginally more prevalent than the hepatocellular (20.0%; 95% CI: 14% to 26%) and mixed patterns (19%; 95% CI: 9% to 30%) (Table 2). In addition, three studies investigated the median time from IMH onset to the resolution, ranging from 7 to 514 days, with an overall pooled mean time of 1.22 months (95% CI: 0.08 to 2.36).

## 4. Discussion

IMH is frequently observed as an AE in cancer individuals undergoing ICI therapy. Despite mild severity, there have been recorded cases of immune-related acute liver failure and even death [11,12]. Additionally, patients with underlying liver diseases were typically excluded due to the challenges in accurately diagnosing IMH and identifying specific biomarkers or symptoms that might distinguish it from the malignancy itself [38]. Consequently, the occurrence and associated risk factors might be underestimated. This meta-analysis included 24 studies with 9076 patients and found the pooled incidence of any grade of IMH [14% (95% CI: 11–17%)] and grade ≥ 3 IMH [7% (95% CI: 5–8%)] in cancer patients. IMH typically developed approximately 2.16 months after initial ICIs and resolved approximately 1.22 months from the onset.

At present, the mechanism of IMH remains unclear. It is well established that PD-L1, as one of the ligands of PD-1, has been detected in the liver. Upon blocking the PD-L1-PD-1 interactions, ICIs trigger the activation of T cells to promote the secretion of various cytokines, including the NLRP3 inflammasome and pro-inflammatory cytokines such as IL-6, IL-1β, IL-18, and tumor necrosis factor, thus resulting in diminished cell viability and causing plasma membrane rupture [39]. Shojaie et al. also demonstrated that ICIs promote dysregulation of adaptive and innate immunity in the liver, resulting in a pro-inflammatory state that hyperactivates both CTLs and myeloid cells. Regions of three-way interaction among cleaved caspase-3+ hepatocytes, CD8+ T-cells, and macrophages were identified in a murine model of IMH using CTLA4+/− mice subjected to either combined immune checkpoint inhibitor treatment with anti-CTLA4 and anti-PD-L1 antibodies or isotype IgG, which subsequently evidenced that hepatocyte apoptosis and Nod-like receptor protein 3 inflammasome activation are crucial in driving IMH [40]. Moreover, ICI treatment can increase gene expression associated with liver cell death [41].

A higher incidence of IMH in younger cancer patients was observed in this study, consistent with a previous meta-analysis [42]. The deterioration of physiological functioning with aging is well established, potentially impacting drug pharmacokinetics, immunological response, and homoeostatic process, thus increasing the risk of AEs [43]. However, the role of age as an independent risk factor for DILI is still a matter of contention primarily due to the limited epidemiological data about DILI in older subjects and the dearth of consistent evidence. Nevertheless, older age was considered a risk factor for certain medications, particularly antimicrobials [44], Chinese herbal drugs [45], and anti-tuberculosis [46]. In contrast, younger individuals are more prone to hepatotoxicity from drugs like valproic acid, minocycline, and salicylates [43]. This phenomenon may be attributed to the diminished functional responsiveness of the innate and adaptive immune systems, characterized by a decrease in B and T cell activation with aging [47]. IMH is defined by the erroneous hyperactivation of the immune system against healthy tissues or organs, while the diminished activity of T cells weakens this excessive immunological response.

Our meta-analysis found that a lower BMI contributed to IMH. Extreme BMI is related to an enhanced risk of death in diverse liver diseases. The connection between BMI and all-cause mortality showed a V-shaped hazard function, with the best prognosis observed at a BIM of 22 kg/m^2^ [48]. However, Ma et al. revealed a J-shaped association between BMI and mortality in DILI; specifically, underweight and obese individuals were considerably associated with higher liver-related mortality [49]. It is speculated that low or high BMI may impact the pharmacokinetics of medications, including their absorption, distribution, metabolism, and excretion [50]. Consequently, individuals with extreme BMI may be more vulnerable to developing severe DILI. Though limited research has examined the impact of BMI on IMH, it is widely considered that malignant tumors severely compromise health, and underweight patients are more prone to being diagnosed at the advanced stage of cancer, rendering them more susceptible to the adverse effects of medications.

Currently, the effect of DM on DILI remains controversial. Freire et al. demonstrated that DM comorbidity was not associated with DILI in patients with tuberculosis [51], while DM was an independent risk factor for DILI in patients with Stevens–Johnson syndrome/toxic epidermal necrolysis or those undergoing therapy with flomoxef [52,53]. To our knowledge, the meta-analysis was the first to recognize DM as a risk factor for IMH, which may be related to the use of antidiabetic drugs. Previous research has indicated that prolonged administration of metformin for type-2 DM typically leads to selective upregulation of H_2_S levels in the liver and, therefore, can result in liver injury [54]. Nevertheless, our study did not include the comorbid medications used during immunotherapy because of missing trial data. Therefore, it is uncertain if the comorbid medicines for DM are responsible for the increased risk of IMH, highlighting the necessity for further investigation.

Pooled data demonstrated a higher risk of IMH in cancer patients receiving ICIs combined with chronic HBV (2.46-fold) and liver metastasis (1.80-fold). Although the gastrointestinal tract, kidney, and skin also exhibit metabolic activities, the liver is primarily responsible for the extensive metabolism of drugs. Cancer patients with chronic HBV or liver metastasis may experience impaired metabolism, hence increasing their vulnerability to toxic substances [9,55]. Furthermore, HBV infection significantly contributes to the progression of HCC and hepatic damage by activating HBV-specific T cells [24]. On the other hand, HBV carriers may exhibit pre-existing liver damage, such as liver fibrosis, and are more prone to experiencing drug-induced hepatotoxicity.

This is the first and most extensive systematic meta-analysis aggregating both crude and adjusted effect sizes to thoroughly assess the significant risk factors of immune-mediated hepatitis secondary to immune checkpoint inhibitors in cancer patients, particularly across various geographical regions, types of immune checkpoint inhibitors, and cancer types. These findings will assist oncologists in making informed decisions on selecting suitable therapies incorporating ICIs and implementing rigorous surveillance during immunotherapy treatment, particularly for patients with recognized risk factors for IMH.

The benefits of our meta-analysis encompass the following key points. Our study comprehensively investigated a wide range of risk factors for IMH, including demographic information, clinical features, previous medical records, and serological biomarkers. These factors were obtained from at least three real-world cohort studies, which is essential because randomized controlled trials typically exclude individuals with pre-existing comorbidities and focus primarily on initial treatment, which can result in underestimating the occurrence of IMH and disregarding certain underlying risk factors. Nevertheless, most studies included were of exceptional quality. In addition, comprehensive subgroup analyses were performed to examine any variations related to geographical regions, types of ICIs, types of cancer, and criteria for diagnosis.

When interpreting the findings, several limitations must be considered. First, the publications examined were mostly retrospective cohort studies, which inherently introduce potential biases due to their design. Second, there was a limited number of studies that provided adjusted ORs for specific risk variables. As a result, the pooled ORs for potential risk factors were derived by converting the baseline characteristics data without excluding the influence of confounding factors. Third, the diagnostic criteria of ICI-IMH varied across studies, and two studies did not report the criteria. This highlights the need for standardized diagnostic criteria for ICI-IMH. Fourth, our meta-analysis primarily aimed to examine the risk factors for IMH rather than focusing on the clinical characteristics of IMH. Several studies were excluded since they did not provide information on risk factors for IMH but did provide data on incidence, patterns, or the resolution time of IMH. Consequently, the results on clinical features of IMH are not broadly applicable. Furthermore, patients with severe IMH (grade III-V) were underrepresented, indicating the necessity for large-scale prospective investigations to further substantiate our findings.

## Figures and Tables

**Table 1:** Characteristics of the studies included in the meta-analysis.

First Author (Year)	Region	Data Source	Study Design	Cancer Type	Criteria of Adverse Events	Tools for Assessing the Causality Between ICIs and Hepatotoxicity	Sample Size/*n*	Gender (Male/Female)	Definition of IMH	IMH Patient/n		Patients Without IMH, *n*
										Any Grade	Grade ≥ 3	
Celsa-1 2024 [15]	Europe, USA, and Asia	Multicenter	Prospective cohort	Multiple	CTCAE 5.0	NA	375	291/84	Grade 1–4	43	16	332
Celsa-2 2024 [15]	Europe, USA, and Asia	Multicenter	Prospective cohort	Multiple	CTCAE 5.0	NA	459	323/136	Grade 1–4	12	5	447
Shen 2024 [16]	China	Monocenter	Retrospective cohort	HCC	CTCAE 5.0	NA	119	109/10	Grade 1–4	52	18	67
Zou 2023 [17]	China	Multicenter	Retrospective cohort	HCC	CSCO guidelines	NA	135	84/51	Grade 1–4	46	8	89
Atallah 2023 [18]	UK	Multicenter	Retrospective case–control	Multiple	CTCAE 5.0	RUCAM	432	NA	Grade 2–4	38	/	394
Jiang 2023 [19]	China	Monocenter	Retrospective cohort	Multiple	Diagnosis and treatment guideline 2017	RUCAM	386	296/92	Grade 1–5	29	/	357
Miah 2023 [20]	USA	Monocenter	Retrospective cohort	Multiple	CTCAE 4.0	NA	1096	649/447	Grade 1–4	64	34	1032
Parlati 2023 [21]	France	Multicenter	Retrospective case–control	Multiple	CTCAE 5.0	RUCAM	952	611/342	Grade 2–4	142	86	810
Zheng 2023 [22]	China	Monocenter	Retrospective cohort	Multiple	CTCAE 5.0	RUCAM	585	450/135	Grade 2–4	71	/	514
Lin 2022 [23]	China	Monocenter	Retrospective cohort	Multiple	CTCAE 5.0	NA	301	215/86	Grade 1–4	51	14	250
Purde 2022 [24]	Switzerland	Monocenter	Prospective cohort	Multiple	CTCAE 5.0	Unclear	84	45/39	Grade 1–4	11	5	73
Smith 2022 [25]	Canada	Multicenter	Retrospective cohort	MM	CTCAE 5.0	NA	63	41/22	Grade 1–4	32	21	31
Swanson 2022 [26]	USA	Monocenter	Retrospective cohort	Multiple	DILIN	DILIN causality score and RUCAM	112	67/45	/	21	/	91
Yamamoto T 2022 [27]	Japan	Multicenter	Retrospective cohort	Lung carcinoma	CTCAE 5.0	NA	365	280/75	Grade 3–4	/	19	346
Biewenga 2021 [28]	Netherlands	Multicenter	Retrospective cohort	MM	CTCAE 4.0	NA	386	231/155	Grade 3–4	80	/	306
Cho 2021 [1]	Korea	Multicenter	Retrospective cohort	Multiple	CTCAE 4.0	NA	194	130/64	Grade 1–4	125	/	69
Cunningham 2021 [29]	Canada	Monocenter	Retrospective cohort	Multiple	CTCAE 5.0	RUCAM	470	240/210	Grade 2–4	17	/	453
Yamamoto A 2021 [30]	Japan	Monocenter	Retrospective cohort	Multiple	CTCAE 5.0	NA	221	177/44	Grade 2–4	21	/	200
Kitagataya 2020 [31]	Japan	Monocenter	Retrospective cohort	Multiple	CTCAE 4.0	DDW-J 2004 scale	202	123/79	Grade 1–4	17	8	185
Li 2020 [32]	China	Multicenter	Retrospective cohort	Multiple	Unclear	NA	112	64/48	Grade 1–5	30	8	82
Mizuno 2020 [33]	Japan	Multicenter	Retrospective cohort	Multiple	CTCAE 4.0	NA	546	397/149	Grade 2–4	44	29	502
Romanski 2020 [34]	Denmark	Monocenter	Retrospective cohort	MM	CTCAE 5.0	NA	637	364/273	Grade 2–4	43	28	594
Sawada 2020 [35]	Japan	Monocenter	Retrospective cohort	Multiple	CTCAE 5.0	DDW-J 2004 scale	135	92/43	Grade 2–4	8	5	127
Tsung 2019 [36]	USA	Monocenter	Retrospective cohort	Multiple	DILIN	RUCAM	491	319/172	/	70	/	421
Huffman 2018 [37]	USA	Monocenter	Retrospective cohort	MM	CTCAE 4.0	NA	218	134/84	Grade 1–4	17	11	201

IMH, immune-mediated hepatotoxicity; HCC, hepatocellular carcinoma; PD-1, anti-programmed death receptor 1; PD-L1, programmed cell death ligand 1; CTLA-4, cytotoxic T-lymphocyte-associated antigen 4; MM, melanoma; DILIN, Drug-Induced Liver Injury Network; RUCAM, Roussel-Uclaf causality assessment method; CTCAE, Common Terminology Criteria for Adverse Event.

**Table 2:** Clinical characteristics for any grade IMH.

Characteristics	No. Study	IMH Patients (n)	Heterogeneity		Pooled Proportion/Mean Value (95% CI)
			*I*^2^ (%)	*p* Value	
Incidence	22	1004	94.9	0.000	14% (95% CI: 11% to 17%)
Hepatocellular pattern	4	37	0.0	0.420	20% (95% CI: 14% to 26%)
Cholestatic pattern	5	107	83.5	0.000	52% (95% CI: 34% to 70%)
Mixed pattern	4	36	67.2	0.027	19% (95% CI: 9% to 30%)
Time to onset of IMH	13	482	0.0	0.962	2.16 months (95% CI: 1.18 to 3.13)
Time to resolution of IMH	3	89	0.0	0.726	1.22 months (95% CI: 0.08 to 2.36)

Abbreviations: IMH, immune-mediated hepatotoxicity; CI, confidence interval.

## Data Availability

The original contributions presented in this study are included in this article/Supplementary Materials. Further inquiries can be directed to the corresponding author.

## Supplementary Materials

The following supporting information can be downloaded at: https://www.mdpi.com/article/10.3390/curroncol31110525/s1. Appendix S1: Search strategy for risk factors; Table S1: Additional immune-related adverse events in patients receiving ICIs; Table S2: Quality assessment of cohort studies using the Newcastle–Ottawa scale; Table S3: Pooled adjusted ORs assessing the risk factors for IMH in patients treated with ICIs; Figure S1: Sensitivity analyses on the source of heterogeneity for age; Figure S2: Sensitivity analyses on the source of heterogeneity for BMI; Figure S3: Sensitivity analyses regarding the source of heterogeneity for the history of ICI treatment; Figure S4: Forest plot of the odds ratio for a series of subgroup analyses regarding any grade of IMH in cancer patients treated with ICIs. (A) Region. (B) data source; (C) cancer type; (D) ICI types; (E) criteria of IMH.
