# Supplementary material for: Risk Factors of Immune-Mediated Hepatotoxicity Induced by Immune Checkpoint Inhibitors in Cancer Patients: A Systematic Review and Meta-Analysis
Source: Curr Oncol. 2024 Nov 13;31(11):7129–43. doi: 10.3390/curroncol31110525 (PMC11593173; doi:10.3390/curroncol31110525)
Supplement: Supplementary file 1 [file curroncol-31-00525-s001.zip › curroncol-3252010-supplementary/Supplementary Appendix S1.pdf]

Appendix S1 Search strategy for risk factors

PubMed/Medline

(((((Immunotherapy[MeSH Terms]) OR (((Immunotherapies[Title/Abstract]) OR (immune Checkpoint Inhibitors[Title/Abstract])) OR (Checkpoint Inhibitors, Immune[Title/Abstract])) OR (Immune Checkpoint Inhibitor[Title/Abstract])) OR (Checkpoint Inhibitor, Immune[Title/Abstract])) OR (Immune Checkpoint Blockers[Title/Abstract])) OR (Checkpoint Blockers, Immune[Title/Abstract])) OR (Immune Checkpoint Blockade[Title/Abstract])) OR (Checkpoint Blockade, Immune[Title/Abstract])) OR (Immune Checkpoint Inhibition[Title/Abstract])) OR (Checkpoint Inhibition, Immune[Title/Abstract])) OR (PD-L1 Inhibitors[Title/Abstract])) OR (PD L1 Inhibitors[Title/Abstract])) OR (PD-L1 Inhibitor[Title/Abstract])) OR (PD L1 Inhibitor[Title/Abstract])) OR (Programmed Death-Ligand 1 Inhibitors[Title/Abstract])) OR (Programmed Death Ligand 1 Inhibitors[Title/Abstract])) OR (PD-1-PD-L1 Blockade[Title/Abstract])) OR (Blockade, PD-1-PD-L1[Title/Abstract])) OR (PD 1 PD L1 Blockade[Title/Abstract])) OR (CTLA-4 Inhibitors[Title/Abstract])) OR (CTLA 4 Inhibitors[Title/Abstract])) OR (CTLA-4 Inhibitor[Title/Abstract])) OR (CTLA 4 Inhibitor[Title/Abstract])) OR (Cytotoxic T-Lymphocyte-Associated Protein 4 Inhibitors[Title/Abstract])) OR (Cytotoxic T Lymphocyte Associated Protein 4 Inhibitors[Title/Abstract])) OR (Cytotoxic T-Lymphocyte-Associated Protein 4 Inhibitor[Title/Abstract])) OR (Cytotoxic T Lymphocyte Associated Protein 4 Inhibitor[Title/Abstract])) OR (PD-1 Inhibitors[Title/Abstract])) OR (PD 1 Inhibitors[Title/Abstract])) OR (PD-1 Inhibitor[Title/Abstract])) OR (Inhibitor, PD-1[Title/Abstract])) OR (PD 1 Inhibitor[Title/Abstract])) OR (Programmed Cell Death Protein 1 Inhibitor[Title/Abstract])) OR (Programmed Cell Death Protein 1 Inhibitor[Title/Abstract])) OR (MEDI0680[Title/Abstract])) OR (MEDI4736[Title/Abstract])) OR (BMS-936558[Title/Abstract])) OR (pembrolizumab[Title/Abstract])) OR (Keytruda[Title/Abstract])) OR (MK-3475[Title/Abstract])) OR (pidilizumab[Title/Abstract])) OR (nivolumab[Title/Abstract])) OR (Opdivo[Title/Abstract])) OR (MDX-1106[Title/Abstract])) OR (ONO-4538[Title/Abstract])) OR (tislelizumab[Title/Abstract])) OR (toripalimab[Title/Abstract])) OR (cemiplimab[Title/Abstract])) OR (sintilimab[Title/Abstract])) OR (SHR-1210[Title/Abstract])) OR (camrelizumab[Title/Abstract])) OR (lambrolizumab[Title/Abstract])) OR (B7 homolog 1[Title/Abstract])) OR (durvalumab[Title/Abstract])) OR (Imfinzi[Title/Abstract])) OR (avelumab[Title/Abstract])) OR (Bavencio[Title/Abstract])) OR (atezolizumab[Title/Abstract])) OR (MPDL3280A[Title/Abstract]))

OR (Tecentriq[Title/Abstract])) OR (RG-7446[Title/Abstract])) OR (ipilimumab[Title/Abstract])) OR (Yervoy[Title/Abstract])) OR (tremelimumab[Title/Abstract]))) AND (((hepatitis[Title/Abstract]) OR (hepatotoxicity[Title/Abstract])) OR (liver injury[Title/Abstract]))) AND (((((((((((((((((((Risk Factors[MeSH Terms]) OR (Factor, Risk[Title/Abstract])) OR (Risk Factor[Title/Abstract])) OR (Social Risk Factors[Title/Abstract])) OR (Factor, Social Risk[Title/Abstract])) OR (Factors, Social Risk[Title/Abstract])) OR (Risk Factor, Social[Title/Abstract])) OR (Risk Factors, Social[Title/Abstract])) OR (Social Risk Factor[Title/Abstract])) OR (Health Correlates[Title/Abstract])) OR (Correlates, Health[Title/Abstract])) OR (Population at Risk[Title/Abstract])) OR (Populations at Risk[Title/Abstract])) OR (Risk Scores[Title/Abstract])) OR (Risk Score[Title/Abstract])) OR (Score, Risk[Title/Abstract])) OR (Risk Factor Scores[Title/Abstract])) OR (Risk Factor Score[Title/Abstract])) OR (Score, Risk Factor[Title/Abstract]))) AND (((((((((((((((((((Risk Factors[MeSH Terms]) OR (Factor, Risk[Title/Abstract])) OR (Risk Factor[Title/Abstract])) OR (Social Risk Factors[Title/Abstract])) OR (Factor, Social Risk[Title/Abstract])) OR (Factors, Social Risk[Title/Abstract])) OR (Risk Factor, Social[Title/Abstract])) OR (Risk Factors, Social[Title/Abstract])) OR (Social Risk Factor[Title/Abstract])) OR (Health Correlates[Title/Abstract])) OR (Correlates, Health[Title/Abstract])) OR (Population at Risk[Title/Abstract])) OR (Populations at Risk[Title/Abstract])) OR (Risk Scores[Title/Abstract])) OR (Risk Score[Title/Abstract])) OR (Score, Risk[Title/Abstract])) OR (Risk Factor Scores[Title/Abstract])) OR (Risk Factor Score[Title/Abstract])) OR (Score, Risk Factor[Title/Abstract]))) AND ((Chemical and Drug Induced Liver Injury[MeSH Terms]) OR (((Chemically-Induced Liver Toxicity[Title/Abstract]) OR (Chemically Induced Liver Toxicity[Title/Abstract])) OR (Chemically-Induced Liver Toxicities[Title/Abstract])) OR (Liver Toxicities, Chemically-Induced[Title/Abstract])) OR (Liver Toxicity, Chemically-Induced[Title/Abstract])) OR (Toxicities, Chemically-Induced Liver[Title/Abstract])) OR (Toxicity, Chemically-Induced Liver[Title/Abstract])) OR (Drug-Induced Acute Liver Injury[Title/Abstract])) OR (Drug Induced Acute Liver Injury[Title/Abstract])) OR (Liver Injury, Drug-Induced, Acute[Title/Abstract])) OR (Acute Liver Injury, Drug-Induced[Title/Abstract])) OR (Acute Liver Injury, Drug Induced[Title/Abstract])) OR (Hepatitis, Toxic[Title/Abstract])) OR (Toxic Hepatitis[Title/Abstract])) OR (Hepatitides, Toxic[Title/Abstract])) OR (Toxic Hepatitides[Title/Abstract])) OR (Drug-Induced Liver Disease[Title/Abstract])) OR (Disease, Drug-Induced Liver[Title/Abstract])) OR (Diseases, Drug-Induced Liver[Title/Abstract])) OR (Drug

Induced Liver Disease[Title/Abstract])) OR (Drug-Induced Liver Diseases[Title/Abstract])) OR (Liver Disease, Drug-Induced[Title/Abstract])) OR (Liver Diseases, Drug-Induced[Title/Abstract])) OR (Drug-Induced Liver Injury[Title/Abstract])) OR (Drug Induced Liver Injury[Title/Abstract])) OR (Drug-Induced Liver Injuries[Title/Abstract])) OR (Injuries, Drug-Induced Liver[Title/Abstract])) OR (Injury, Drug-Induced Liver[Title/Abstract])) OR (Liver Injuries, Drug-Induced[Title/Abstract])) OR (Liver Injury, Drug-Induced[Title/Abstract])) OR (Liver Injury, Drug Induced[Title/Abstract])) OR (Hepatitis, Drug-Induced[Title/Abstract])) OR (Drug-Induced Hepatitides[Title/Abstract])) OR (Drug-Induced Hepatitis[Title/Abstract])) OR (Hepatitides, Drug-Induced[Title/Abstract])) OR (Hepatitis, Drug Induced[Title/Abstract])) OR (Hepatic Insufficiency[Title/Abstract])) OR (Insufficiency, Hepatic[Title/Abstract])) OR (Liver Insufficiency[Title/Abstract])) OR (Insufficiency, Liver[Title/Abstract])) OR (Hepatotoxicity[Title/Abstract])))

Cochrane

ID Search Hits

#1 MeSH descriptor: [Immunotherapy] explode all trees 12047

#2 (Immunotherapy):ti,ab,kw OR (Immunotherapies):ti,ab,kw OR (immune Checkpoint Inhibitors):ti,ab,kw OR (Checkpoint Inhibitors, Immune):ti,ab,kw OR (Immune Checkpoint Inhibitor):ti,ab,kw 14885

#3 (Checkpoint Inhibitor, Immune):ti,ab,kw OR (Immune Checkpoint Blockers):ti,ab,kw OR (Checkpoint Blockers, Immune):ti,ab,kw OR (Immune Checkpoint Blockade):ti,ab,kw OR (Checkpoint Blockade, Immune):ti,ab,kw 1397

#4 (Immune Checkpoint Inhibition):ti,ab,kw OR (Checkpoint Inhibition, Immune):ti,ab,kw OR (PD-L1 Inhibitors):ti,ab,kw OR (PD L1 Inhibitors):ti,ab,kw OR (PD-L1 Inhibitor):ti,ab,kw 1918

#5 (PD L1 Inhibitor):ti,ab,kw OR (Programmed Death-Ligand 1 Inhibitors):ti,ab,kw OR (Programmed Death Ligand 1 Inhibitors):ti,ab,kw OR (PD 1 PD L1 Blockade):ti,ab,kw OR (CTLA-4 Inhibitors):ti,ab,kw 1602

#6 (CTLA 4 Inhibitors):ti,ab,kw OR (CTLA-4 Inhibitor):ti,ab,kw OR (CTLA 4 Inhibitor):ti,ab,kw OR (Cytotoxic T-Lymphocyte-Associated Protein 4 Inhibitors):ti,ab,kw OR (Cytotoxic T Lymphocyte Associated Protein 4 Inhibitors):ti,ab,kw 353

#7 (Cytotoxic T-Lymphocyte-Associated Protein 4 Inhibitor):ti,ab,kw OR (Cytotoxic T Lymphocyte Associated Protein 4 Inhibitor):ti,ab,kw OR (PD-1 Inhibitors):ti,ab,kw OR (PD 1 Inhibitors):ti,ab,kw

OR (PD 1 Inhibitors):ti,ab,kw 3922

#8 (PD 1 Inhibitors):ti,ab,kw OR (PD 1 Inhibitor):ti,ab,kw OR (Programmed Cell Death Protein 1 Inhibitor):ti,ab,kw OR (Programmed Cell Death Protein 1 Inhibitors):ti,ab,kw OR (MEDI0680):ti,ab,kw 6772

#9 (MEDI4736):ti,ab,kw OR (BMS-936558):ti,ab,kw OR (pembrolizumab):ti,ab,kw OR (Keytruda):ti,ab,kw OR (MK-3475):ti,ab,kw 3488

#10 (pidilizumab):ti,ab,kw OR (nivolumab):ti,ab,kw OR (Opdivo):ti,ab,kw OR (MDX-1106):ti,ab,kw OR (ONO-4538):ti,ab,kw 3028

#11 (tislelizumab):ti,ab,kw OR (toripalimab):ti,ab,kw OR (cemiplimab):ti,ab,kw OR (sintilimab):ti,ab,kw OR (SHR-1210):ti,ab,kw 814

#12 (SHR-1210):ti,ab,kw OR (lambrolizumab):ti,ab,kw OR (B7 homolog 1):ti,ab,kw OR (durvalumab):ti,ab,kw OR (Imfinzi):ti,ab,kw 1223

#13 (avelumab):ti,ab,kw OR (Bavencio):ti,ab,kw OR (atezolizumab):ti,ab,kw OR (MPDL3280A):ti,ab,kw OR (Tecentriq):ti,ab,kw 1826

#14 (RG-7446):ti,ab,kw OR (ipilimumab):ti,ab,kw OR (Yervoy):ti,ab,kw OR (tremelimumab):ti,ab,kw 2266

#15 #1 or #2 or #3 or #4 or #5 or #6 or #7 or #8 or #9 or #10 or #11 or #12 or #13 or #14 34473

#16 MeSH descriptor: [Risk Factors] explode all trees 38033

#17 (Risk Factors):ti,ab,kw OR (Factor, Risk):ti,ab,kw OR (Risk Factor):ti,ab,kw OR (Social Risk Factors):ti,ab,kw OR (Factor, Social Risk):ti,ab,kw 123538

#18 (Factors, Social Risk):ti,ab,kw OR (Risk Factor, Social):ti,ab,kw OR (Risk Factors, Social):ti,ab,kw OR (Social Risk Factor):ti,ab,kw OR (Health Correlates):ti,ab,kw 8570

#19 (Correlates, Health):ti,ab,kw OR (Population at Risk):ti,ab,kw OR (Populations at Risk):ti,ab,kw OR (Risk Scores):ti,ab,kw OR (Risk Score):ti,ab,kw98988

#20 (Score, Risk):ti,ab,kw OR (Risk Factor Scores):ti,ab,kw OR (Risk Factor Score):ti,ab,kw OR (Score, Risk Factor):ti,ab,kw OR (Cause):ti,ab,kw 116521

#21 (Predictive factor):ti,ab,kw 5550

#22 #16 or #17 or #18 or #19 or #20 or #21 244370

#23 MeSH descriptor: [Chemical and Drug Induced Liver Injury] explode all trees 474

#24 (Chemical and Drug Induced Liver Injury):ti,ab,kw OR (Chemically-Induced Liver Toxicity):ti,ab,kw OR (Chemically Induced Liver Toxicity):ti,ab,kw OR (Chemically-Induced Liver

Toxicities):ti,ab,kw OR (Liver Toxicities, Chemically-Induced):ti,ab,kw 779

#25 (Liver Toxicity, Chemically-Induced):ti,ab,kw OR (Toxicities, Chemically-Induced Liver):ti,ab,kw OR (Toxicity, Chemically-Induced Liver):ti,ab,kw OR (Drug-Induced Acute Liver Injury):ti,ab,kw OR (Drug Induced Acute Liver Injury):ti,ab,kw 582

#26 (Liver Injury, Drug-Induced, Acute):ti,ab,kw OR (Acute Liver Injury, Drug-Induced):ti,ab,kw OR (Acute Liver Injury, Drug Induced):ti,ab,kw OR (Hepatitis, Toxic):ti,ab,kw OR (Toxic Hepatitis):ti,ab,kw 562

#27 (Hepatitides, Toxic):ti,ab,kw OR (Toxic Hepatitides):ti,ab,kw OR (Drug-Induced Liver Disease):ti,ab,kw OR (Disease, Drug-Induced Liver):ti,ab,kw OR (Diseases, Drug-Induced Liver):ti,ab,kw 635

#28 (Drug Induced Liver Disease):ti,ab,kw OR (Drug-Induced Liver Diseases):ti,ab,kw OR (Liver Disease, Drug-Induced):ti,ab,kw OR (Liver Diseases, Drug-Induced):ti,ab,kw OR (Drug-Induced Liver Injury):ti,ab,kw2189

#29 (Drug Induced Liver Injury):ti,ab,kw OR (Drug-Induced Liver Injuries):ti,ab,kw OR (Injuries, Drug-Induced Liver):ti,ab,kw OR (Injury, Drug-Induced Liver):ti,ab,kw OR (Liver Injuries, Drug-Induced):ti,ab,kw 978

#30 (Liver Injury, Drug-Induced):ti,ab,kw OR (Liver Injury, Drug Induced):ti,ab,kw OR (Hepatitis, Drug-Induced):ti,ab,kw OR (Drug-Induced Hepatitides):ti,ab,kw OR (Drug-Induced Hepatitis):ti,ab,kw 1169

#31 (Hepatitides, Drug-Induced):ti,ab,kw OR (Hepatitides, Drug-Induced):ti,ab,kw OR (Hepatic Insufficiency):ti,ab,kw OR (Insufficiency, Hepatic):ti,ab,kw OR (Liver Insufficiency):ti,ab,kw 1182

#32 (Insufficiency, Liver):ti,ab,kw OR (Hepatotoxicity):ti,ab,kw 2006

#33 #23 or #24 or #25 or #26 or #27 or #28 or #29 or #30 or #31 or #32 4706

#34 #15 AND #22 AND #33 45

Web of science

1# TS=(Immunotherapy) OR TS=(Immunotherapies) OR TS=(immune Checkpoint Inhibitors) OR TS=(Checkpoint Inhibitors, Immune) OR TS=(Immune Checkpoint Inhibitor) OR TS=(Checkpoint Inhibitor, Immune) OR TS=(Immune Checkpoint Blockers) OR TS=(Checkpoint Blockers, Immune) OR TS=(Immune Checkpoint Blockade) OR TS=(Checkpoint Blockade, Immune) OR TS=(Immune Checkpoint Inhibition) OR TS=(Checkpoint Inhibition, Immune) OR TS=(PD-L1 Inhibitors) OR

TS=(PD L1 Inhibitors) OR TS=(PD-L1 Inhibitor) OR TS=(PD L1 Inhibitor) OR TS=(Programmed Death-Ligand 1 Inhibitors) OR TS=(Programmed Death Ligand 1 Inhibitors) OR TS=(PD-1-PD-L1 Blockade) OR TS=(Blockade, PD-1-PD-L1) OR TS=(PD 1 PD L1 Blockade) OR TS=(CTLA-4 Inhibitors) OR TS=(CTLA 4 Inhibitors) OR TS=(CTLA-4 Inhibitor) OR TS=(CTLA 4 Inhibitor) OR TS=(Cytotoxic T-Lymphocyte-Associated Protein 4 Inhibitors) OR TS=(Cytotoxic T Lymphocyte Associated Protein 4 Inhibitors) OR TS=(Cytotoxic T-Lymphocyte-Associated Protein 4 Inhibitor) OR TS=(Cytotoxic T Lymphocyte Associated Protein 4 Inhibitor) OR TS=(PD-1 Inhibitors) OR TS=(PD 1 Inhibitors) OR TS=(PD-1 Inhibitor) OR TS=(Inhibitor, PD-1) OR TS=(PD 1 Inhibitor) OR TS=(Programmed Cell Death Protein 1 Inhibitor) OR TS=(Programmed Cell Death Protein 1 Inhibitors) OR TS=(MEDI0680) OR TS=(MEDI4736) OR TS=(BMS-936558) OR TS=(pembrolizumab) OR TS=(Keytruda) OR TS=(MK-3475) OR TS=(pidilizumab) OR TS=(nivolumab) OR TS=(Opdivo) OR TS=(MDX-1106) OR TS=(ONO-4538) OR TS=(tislelizumab) OR TS=(toripalimab) OR TS=(cemiplimab) OR TS=(sintilimab) OR TS=(SHR-1210) OR TS=(camrelizumab) OR TS=(lambrolizumab) OR TS=(B7 homolog 1) OR TS=(durvalumab) OR TS=(Imfinzi) OR TS=(avelumab) OR TS=(Bavencio) OR TS=(atezolizumab) OR TS=(MPDL3280A) OR TS=(Tecentriq) OR TS=(RG-7446) OR TS=(ipilimumab) OR TS=(Yervoy) OR TS=(tremelimumab)

233072

2# TS=(Risk Factors) OR TS=(Factor, Risk) OR TS=(Risk Factor) OR TS=(Social Risk Factors) OR TS=(Factor, Social Risk) OR TS=(Factors, Social Risk) OR TS=(Risk Factor, Social) OR TS=(Risk Factors, Social) OR TS=(Social Risk Factor) OR TS=(Health Correlates) OR TS=(Correlates, Health) OR TS=(Population at Risk) OR TS=(Populations at Risk) OR TS=(Risk Scores) OR TS=(Risk Score) OR TS=(Score, Risk) OR TS=(Risk Factor Scores) OR TS=(Risk Factor Score) OR TS=(Score, Risk Factor) OR TS=(Cause) OR TS=(Predictive factor) 5742732

3# TS=(Chemical and Drug Induced Liver Injury) OR TS=(Chemically-Induced Liver Toxicity) OR TS=(Chemically Induced Liver Toxicity) OR TS=(Chemically-Induced Liver Toxicities) OR TS=(Liver Toxicities, Chemically-Induced) OR TS=(Liver Toxicity, Chemically-Induced) OR TS=(Toxicities, Chemically-Induced Liver) OR TS=(Toxicity, Chemically-Induced Liver) OR TS=(Drug-Induced Acute Liver Injury) OR TS=(Drug Induced Acute Liver Injury) OR TS=(Liver Injury, Drug-Induced, Acute) OR TS=(Acute Liver Injury, Drug-Induced) OR TS=(Acute Liver Injury, Drug Induced) OR TS=(Hepatitis, Toxic) OR TS=(Toxic Hepatitis) OR TS=(Hepatitides, Toxic) OR TS=(Toxic Hepatitides) OR TS=(Drug-Induced Liver Disease) OR TS=(Disease, Drug-Induced Liver)

OR TS=(Diseases, Drug-Induced Liver) OR TS=(Drug Induced Liver Disease) OR TS=(Drug-Induced Liver Diseases) OR TS=(Liver Disease, Drug-Induced) OR TS=(Liver Diseases, Drug-Induced) OR TS=(Drug-Induced Liver Injury) OR TS=(Drug Induced Liver Injury) OR TS=(Drug-Induced Liver Injuries) OR TS=(Injuries, Drug-Induced Liver) OR TS=(Injury, Drug-Induced Liver) OR TS=(Liver Injuries, Drug-Induced) OR TS=(Liver Injury, Drug-Induced) OR TS=(Liver Injury, Drug Induced) OR TS=(Hepatitis, Drug-Induced) OR TS=(Drug-Induced Hepatitides) OR TS=(Drug-Induced Hepatitis) OR TS=(Hepatitides, Drug-Induced) OR TS=(Hepatitis, Drug Induced) OR TS=(Hepatic Insufficiency) OR TS=(Insufficiency, Hepatic) OR TS=(Liver Insufficiency) OR TS=(Insufficiency, Liver) OR TS=(Hepatotoxicity) 55408

3# #3 AND #2 AND #1 228

Embase

#134 #65 AND #88 AND #133 173 Date 29-Mar-24

#133 #89 OR #90 OR #91 OR #92 OR #93 OR #94 OR #95 OR #96 OR #97 OR #98 OR #99 OR #100 OR #101 OR #102 OR #103 OR #104 OR #105 OR #106 OR #107 OR #108 OR #109 OR #110 OR #111 OR #112 OR #113 OR #114 OR #115 OR #116 OR #117 OR #118 OR #119 OR #120 OR #121 OR #122 OR #123 OR #124 OR #125 OR #126 OR #127 OR #128 OR #129 OR #130 OR #131 47122

#131 'hepatotoxicity':ab,ti 34675

#130 'insufficiency, liver':ab,ti 105

#129 'liver insufficiency':ab,ti 1249

#128 'insufficiency, hepatic':ab,ti 78

#127 'hepatic insufficiency':ab,ti 2304

#126 'hepatitis, drug induced':ab,ti 72

#125 'hepatitides, drug-induced':ab,ti 0

#124 'drug-induced hepatitis':ab,ti 943

#123 'drug-induced hepatitides':ab,ti 1

#122 'hepatitis, drug-induced':ab,ti 72

#121 'liver injury, drug-induced':ab,ti 30

#120 'liver injury, drug-induced':ab,ti 30

#119 'liver injuries, drug-induced':ab,ti 1

#118 'injury, drug-induced liver':ab,ti 30

#117 'injuries, drug-induced liver':ab,ti 1

#116 'drug-induced liver injuries':ab,ti 122

#115 'drug induced liver injury':ab,ti 6953

#114 'drug-induced liver injury':ab,ti 6973

#113 'liver diseases, drug-induced':ab,ti 6

#112 'liver disease, drug-induced':ab,ti 42

#111 'drug-induced liver diseases':ab,ti 70

#110 'drug induced liver disease':ab,ti 330

#109 'diseases, drug-induced liver':ab,ti 7

#108 'disease, drug-induced liver':ab,ti 44

#107 'drug-induced liver disease':ab,ti 330

#106 'toxic hepatitides':ab,ti 1

#105 'hepatitides, toxic':ab,ti 0

#104 'toxic hepatitis':ab,ti 1501

#103 'hepatitis, toxic':ab,ti 45

#102 'acute liver injury, drug-induced':ab,ti 2

#101 'acute liver injury, drug-induced':ab,ti 2

#100 'liver injury, drug-induced, acute':ab,ti 1

#99 'drug induced acute liver injury':ab,ti 49

#98 'drug-induced acute liver injury':ab,ti 49

#97 'toxicity, chemically-induced liver':ab,ti 0

#96 'toxicities, chemically-induced liver':ab,ti 0

#95 'liver toxicity, chemically-induced':ab,ti 0

#94 'liver toxicities, chemically-induced':ab,ti 0

#93 'chemically-induced liver toxicities':ab,ti 0

#92 'chemically induced liver toxicity':ab,ti 8

#91 'chemically-induced liver toxicity':ab,ti 8

#90 'chemical and drug induced liver injury':ab,ti 5

#89 'chemical and drug induced liver injury'/exp 3396

#88 #66 OR #67 OR #68 OR #69 OR #70 OR #71 OR #72 OR #73 OR #74 OR #75 OR #76 OR #77

OR #78 OR #79 OR #80 OR #81 OR #82 OR #83 OR #84 OR #85 OR #86 OR #87 3486602

#87 'predictive factor':ab,ti 18262

#86 'cause':ab,ti 1771224

#85 'score, risk factor':ab,ti 17

#84 'risk factor score':ab,ti 297

#83 'risk factor scores':ab,ti 146

#82 'score, risk':ab,ti 1351

#81 'risk score':ab,ti 49739

#80 'risk scores':ab,ti 23379

#79 'populations at risk':ab,ti 3706

#78 'population at risk':ab,ti 5994

#77 'correlates, health':ab,ti 12

#76 'health correlates':ab,ti 628

#75 'social risk factor':ab,ti 114

#74 'risk factors, social':ab,ti 148

#73 'risk factor, social':ab,ti 7

#72 'factors, social risk':ab,ti 9

#71 'factor, social risk':ab,ti 0

#70 'social risk factors':ab,ti 1430

#69 'risk factor':ab,ti 409728

#68 'factor, risk':ab,ti 345

#67 'risk factors':ab,ti 880464

#66 'risk factor'/exp 1411041

#65 #1 OR #2 OR #3 OR #4 OR #5 OR #6 OR #7 OR #8 OR #9 OR #10 OR #11 OR #12 OR #13 OR #14 OR #15 OR #16 OR #17 OR #18 OR #19 OR #20 OR #21 OR #22 OR #23 OR #24 OR #25 OR #26 OR #27 OR #28 OR #29 OR #30 OR #31 OR #32 OR #33 OR #34 OR #35 OR #36 OR #37 OR #38 OR #39 OR #40 OR #41 OR #42 OR #43 OR #44 OR #45 OR #46 OR #47 OR #48 OR #49 OR #50 OR #51 OR #52 OR #53 OR #54 OR #55 OR #56 OR #57 OR #58 OR #59 OR #60 OR #61 OR #62 OR #63 OR #64 392573

#64 'tremelimumab':ab,ti 1174

#63 'yervoy':ab,ti 126

#62 'ipilimumab':ab,ti 11303

#61 'rg-7446':ab,ti 0

#60 'tecentriq':ab,ti 79

#59 'mpdl3280a':ab,ti 129

#58 'atezolizumab':ab,ti 7102

#57 'bavencio':ab,ti 19

#56 'avelumab':ab,ti 2158

#55 'imfinzi':ab,ti 34

#54 'durvalumab':ab,ti 4268

#53 'b7 homolog 1':ab,ti 48

#52 'lambrolizumab':ab,ti 28

#51 'camrelizumab':ab,ti 969

#50 'shr-1210':ab,ti 85

#49 'sintilimab':ab,ti 917

#48 'cemiplimab':ab,ti 860

#47 'toripalimab':ab,ti 585

#46 'tislelizumab':ab,ti 753

#45 'ono-4538':ab,ti 135

#44 'mdx-1106':ab,ti 31

#43 'opdivo':ab,ti 177

#42 'nivolumab':ab,ti 20951

#41 'pidilizumab':ab,ti 37

#40 'mk-3475':ab,ti 261

#39 'keytruda':ab,ti 337

#38 'pembrolizumab':ab,ti 20162

#37 'bms-936558':ab,ti 141

#36 'medi4736':ab,ti 258

#35 'medi0680':ab,ti 17

#34 'programmed cell death protein 1 inhibitors':ab,ti 66

#33 'programmed cell death protein 1 inhibitors':ab,ti 66

#32 'programmed cell death protein 1 inhibitor':ab,ti 101

#31 'pd 1 inhibitor':ab,ti 3288

#30 'inhibitor, pd-1':ab,ti 78

#29 'pd-1 inhibitor':ab,ti 3290

#28 'pd-1 inhibitors':ab,ti 2786

#27 'pd-1 inhibitors':ab,ti 2786

#26 'cytotoxic t lymphocyte associated protein 4 inhibitor':ab,ti 20

#25 'cytotoxic t-lymphocyte-associated protein 4 inhibitor':ab,ti 20

#24 'cytotoxic t lymphocyte associated protein 4 inhibitors':ab,ti 22

#23 'cytotoxic t-lymphocyte-associated protein 4 inhibitors':ab,ti 22

#22 'ctla 4 inhibitor':ab,ti 377

#21 'ctla-4 inhibitor':ab,ti 377

#20 'ctla 4 inhibitors':ab,ti 466

#19 'ctla-4 inhibitors':ab,ti 466

#18 'pd 1 pd l1 blockade':ab,ti 1069

#17 'blockade, pd-1-pd-l1':ab,ti 5

#16 'pd-1-pd-l1 blockade':ab,ti 1069

#15 'programmed death ligand 1 inhibitors':ab,ti 97

#14 'programmed death-ligand 1 inhibitors':ab,ti 99

#13 'pd-l1 inhibitor':ab,ti 1650

#12 'pd-l1 inhibitors':ab,ti 2721

#11 'checkpoint inhibition, immune':ab,ti 4

#10 'checkpoint blockade, immune':ab,ti 12

#9 'immune checkpoint blockade':ab,ti 10066

#8 'checkpoint blockers, immune':ab,ti 0

#7 'immune checkpoint blockers':ab,ti 684

#6 'checkpoint inhibitor, immune':ab,ti 15

#5 'immune checkpoint inhibitor':ab,ti 11622

#4 'checkpoint inhibitors, immune':ab,ti 53

#3 'immune checkpoint inhibitors':ab,ti 31629

#2 'immunotherapies':ab,ti 25962

#1 'immunotherapy'/exp 332597
