# Supplementary material for: Risk Factors of Immune-Mediated Hepatotoxicity Induced by Immune Checkpoint Inhibitors in Cancer Patients: A Systematic Review and Meta-Analysis
Source: Curr Oncol. 2024 Nov 13;31(11):7129–43. doi: 10.3390/curroncol31110525 (PMC11593173; doi:10.3390/curroncol31110525)

Supplementary Figure S1 Sensitivity analyses on the source of heterogeneity for age.

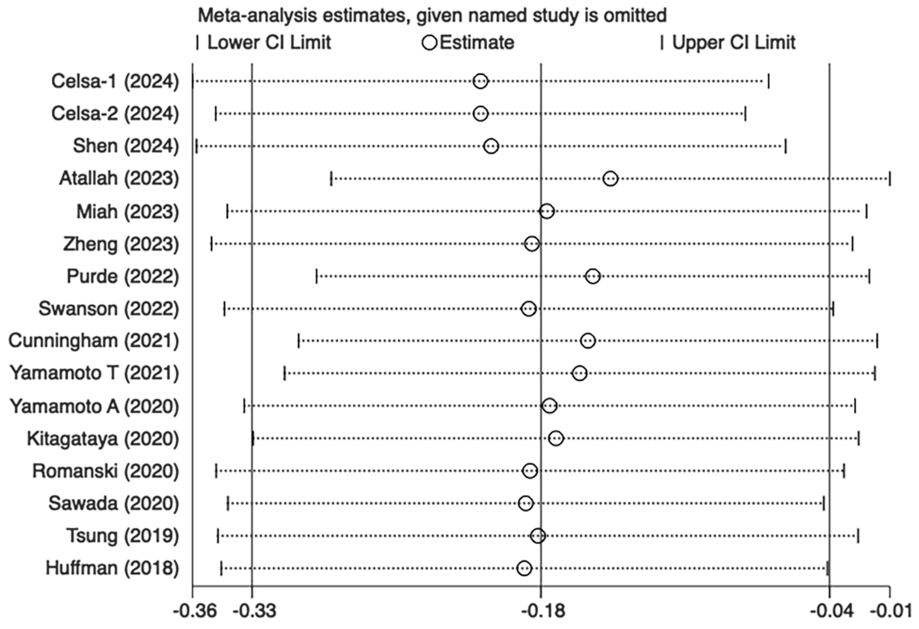

Supplementary Figure S2 Sensitivity analyses on the source of heterogeneity for BMI.

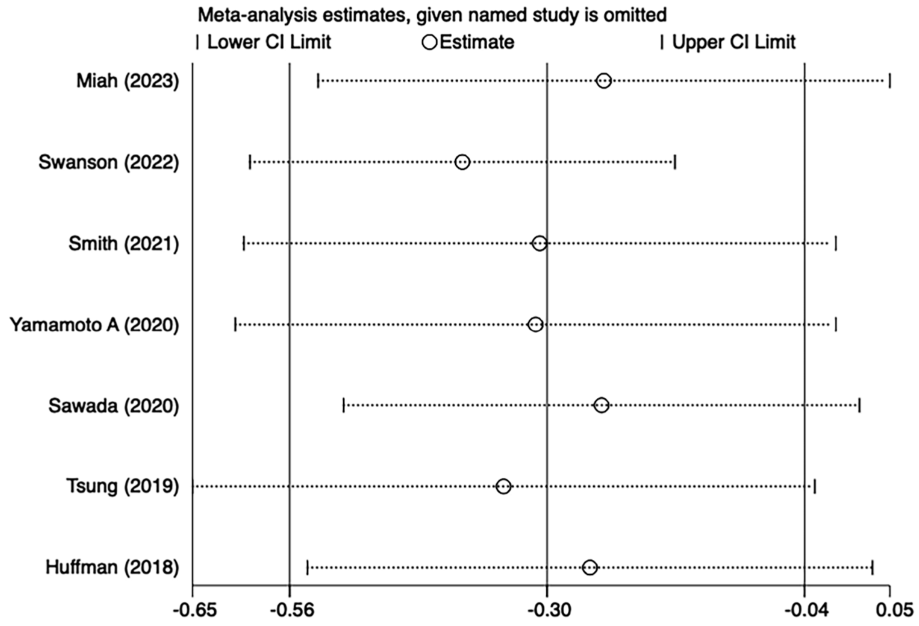

Supplementary Figure S3 Sensitivity analyses regarding the source of heterogeneity for history of ICI treatment.

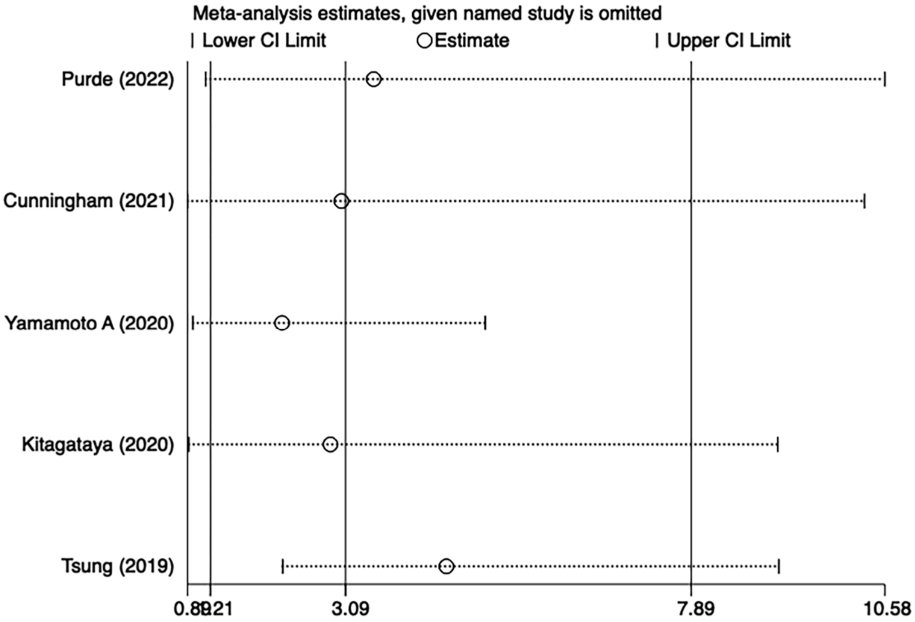

Supplementary Figure S4 Forest plot of the odds ratio for a series of subgroup analyses regarding any grade of IMH in cancer patients treated with ICIs. (A) Region. (B) Data source. (C) Cancer type. (D) ICI types. (E) Criteria of IMH. The blue rhombus signifies the pooled effect size.

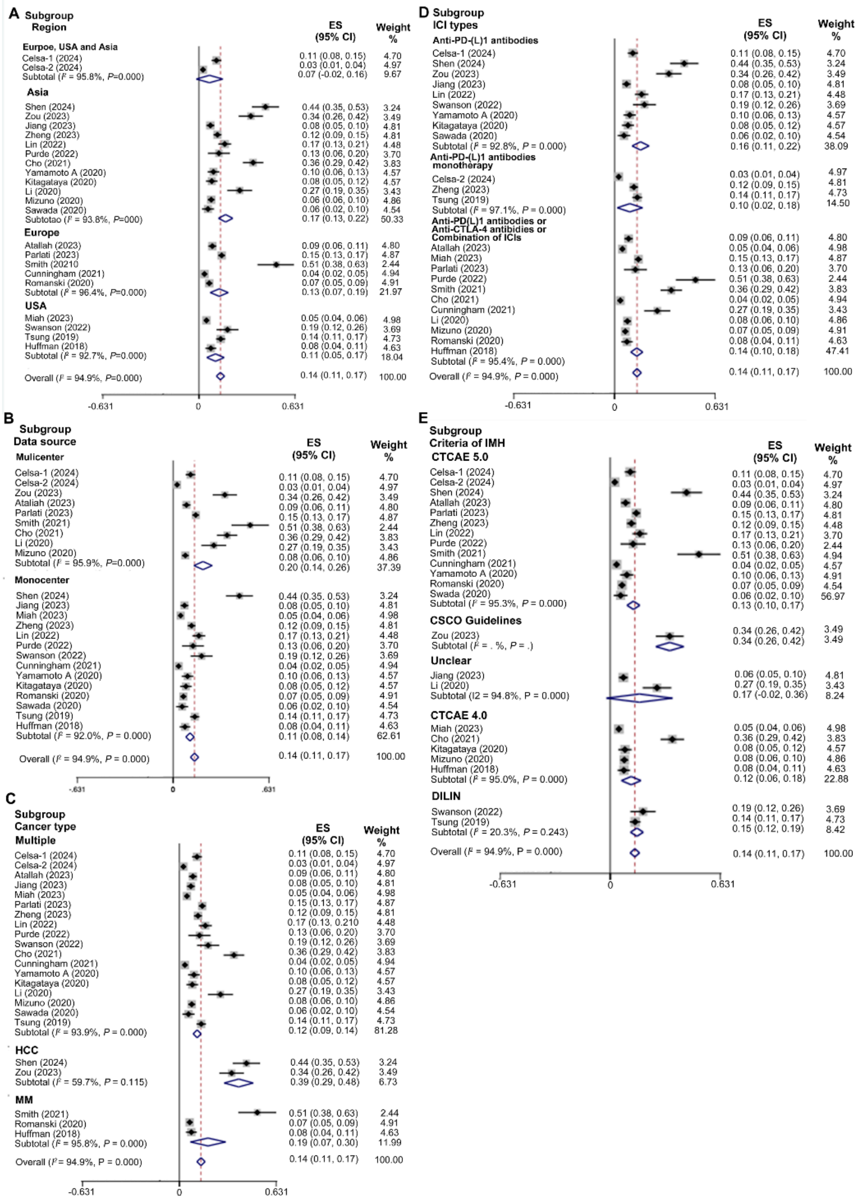

Supplement: Supplementary file 1 [file curroncol-31-00525-s001.zip › curroncol-3252010-supplementary/Supplementary Figures .pdf]
