# Supplementary material for: Risk Factors of Immune-Mediated Hepatotoxicity Induced by Immune Checkpoint Inhibitors in Cancer Patients: A Systematic Review and Meta-Analysis
Source: Curr Oncol. 2024 Nov 13;31(11):7129–43. doi: 10.3390/curroncol31110525 (PMC11593173; doi:10.3390/curroncol31110525)
Supplement: Supplementary file 1 [file curroncol-31-00525-s001.zip › curroncol-3252010-supplementary/Table S1.pdf]

Supplementary Table S1. Additional immune-related adverse events in patients receiving ICIs.

IrAE	Total, n(%)	Reference
Gastrointestinal	42 (11.2)	Celsa-1 2024 [15]
Endocrine	11 (2.9)	Celsa-1 2024 [15]
Skin	37 (9.9)	Celsa-1 2024 [15]
Lung	3 (0.8)	Celsa-1 2024 [15]
Neuromuscolar and rheumatologic	14 (3.7)	Celsa-1 2024 [15]
Gastrointestinal	47 (10.2)	Celsa-2 2024 [15]
Endocrine	41 (8.9)	Celsa-2 2024 [15]
Skin	58 (12.6)	Celsa-2 2024 [15]
Lung	15 (3.3)	Celsa-2 2024 [15]
Neuromuscolar and rheumatologic	27 (5.9)	Celsa-2 2024 [15]
**Concurrent irAE**	**Total, n(%)**	**Reference**
Thyroiditis	20(20.2)	Atallah 2023 [18]
Colitis	20(20.2)	Atallah 2023 [18]
Skin rash	14(14.1)	Atallah 2023 [18]
Adrenalitis	9(9.1)	Atallah 2023 [18]
Hypophysitis	5(5.1)	Atallah 2023 [18]
Skin	7(63.6)	Purde 2022 [24]
Gastrointestinal	3(27.3)	Purde 2022 [24]
Peripheral nervous system	1(9.1)	Purde 2022 [24]
Endocrine	1(9.1)	Purde 2022 [24]
Kidney	1(9.1)	Purde 2022 [24]
Lung	1(9.1)	Purde 2022 [24]
Enterocolitis/diarrhoea	26(41.3)	Smith 2022 [25]
Other irAEs	34(53.9)	Smith 2022 [25]
Endocrine	17(12.2)	Biewenga 2021 [28]
Gastrointestinal	13(9.4)	Biewenga 2021 [28]
Skin	10(7.2)	Biewenga 2021 [28]
Respiratory	5(3.6)	Biewenga 2021 [28]
Blood	2(1.4)	Biewenga 2021 [28]
Neurological	2(1.4)	Biewenga 2021 [28]
Fatigue	1(0.7)	Biewenga 2021 [28]
Endocrine	3(23.1)	Cunningham 2021 [29]
Skin	3(23.1)	Cunningham 2021 [29]
Gastrointestinal	4(30.8)	Cunningham 2021 [29]
Respiratory	3(23.1)	Cunningham 2021 [29]
Renal	1(7.7)	Cunningham 2021 [29]
CNS	1(7.7)	Cunningham 2021 [29]

Musculoskeletal	2(15.4)	Cunningham 2021 [29]
Haematology	1(7.7)	Cunningham 2021 [29]
Pancreatitis*	1(8.3)	Yamamoto T 2022 [27]
Colitis*	1(8.3)	Yamamoto T 2022 [27]
Thyroid dysfunction*	1(8.3)	Yamamoto T 2022 [27]
Pancreatitis	6(28.6)	Yamamoto A 2021 [30]
Nephritis	1(4.8)	Yamamoto A 2021 [30]
Myasthenia	1(4.8)	Yamamoto A 2021 [30]
Hyperthyroidism	1(4.8)	Yamamoto A 2021 [30]
Rash	1(4.8)	Yamamoto A 2021 [30]
Colitis	1(4.8)	Yamamoto A 2021 [30]
Colitis*	3(10.3)	Mizuno 2020 [33]
Pneumonitis*	1(3.4)	Mizuno 2020 [33]
Colitis	2(11.7)	Huffman 2018 [37]
Hypophysitis	2(11.7)	Huffman 2018 [37]
Rash	2(11.7)	Huffman 2018 [37]
Pneumonitis	2(11.7)	Huffman 2018 [37]

IrAEs: Immune-related adverse events; ICIs: Immune checkpoint inhibitors; CNS: Central Nervous System

*Patients with severe immune-related liver injury (Grade 3)
