# Supplementary material for: Risk Factors of Immune-Mediated Hepatotoxicity Induced by Immune Checkpoint Inhibitors in Cancer Patients: A Systematic Review and Meta-Analysis
Source: Curr Oncol. 2024 Nov 13;31(11):7129–43. doi: 10.3390/curroncol31110525 (PMC11593173; doi:10.3390/curroncol31110525)
Supplement: Supplementary file 1 [file curroncol-31-00525-s001.zip › curroncol-3252010-supplementary/Table S2.pdf]

Table S2. Quality assessment of cohort studies using the Newcastle-Ottawa scale

Cohort study									
	Selection				Comparability	Outcome			
Study	Representativeness of the exposed group	Selection of non-exposed groups	Determination of exposure factors	Identification of outcome indicators not yet to be observed at study entry	Comparability of exposed and unexposed groups considered in design and statistical analysis	Design and statistical analysis	Adequacy of the study's evaluation of the outcome	Adequacy of follow-up in exposed and unexposed groups	Total scores
Celsa-1 2024	1	1	1	1	1	1	1	1	8
Celsa-2 2024	1	1	1	1	1	1	1	1	8
Shen 2023	1	1	1	1	2	1	1	1	9
Zou 2023	1	1	1	1	1	1			6
Atallah 2023	1	1	1	1	1	1	1	1	8
Jiang 2023	1	1			2	1	1	1	7
Miah 2023	1	1	1		1	1	1		6
Parlati	1	1	1	1	2	1	1	1	9

2023									
Zheng 2023	1	1	1	1	1	1			6
Lin 2022	1	1	1	1	2	1			7
Purde 2022	1	1	1	1	1	1	1	1	8
Smith 2022	1	1	1	1	1	1			6
Swanson 2022	1	1	1	1	1	1			6
Biewenga 2021	1	1	1	1	1	1	1		7
Cho 2021	1	1	1	1	1	1			6
Cunningham 2021	1	1	1	1		1	1	1	7
Yamamoto T 2021	1	1	1	1	1	1	1	1	8
Yamamoto A 2020	1	1	1	1	2	1	1	1	9
Kitagataya 2020	1	1	1	1	1	1	1	1	8
Li	1	1	1	1	1	1			6

2020									
Mizuno 2020	1	1	1	1	1	1	1	1	8
Romanski 2020	1	1	1	1	1	1	1	1	8
Sawada 2020	1	1	1	1	2	1	1	1	9
Tsung 2019	1	1	1	1		1	1	1	7
Huffman 2018	1	1	1	1	1	1		1	7
