# Supplementary material for: Risk Factors of Immune-Mediated Hepatotoxicity Induced by Immune Checkpoint Inhibitors in Cancer Patients: A Systematic Review and Meta-Analysis
Source: Curr Oncol. 2024 Nov 13;31(11):7129–43. doi: 10.3390/curroncol31110525 (PMC11593173; doi:10.3390/curroncol31110525)
Supplement: Supplementary file 1 [file curroncol-31-00525-s001.zip › curroncol-3252010-supplementary/Table S3.pdf]

Supplementary Table S3 Pooled adjusted ORs assessing the risk factors for IMH in patients treated with ICIs

Factors	No. study	Heterogeneity test		OR (95% CI)	*P* value	Egger's test
		I^2(%)	*P* value			
**Gender (Male)**	3	0	0.478	0.44 (0.27, 0.72)	0.001	0.145
Combination of ICIs	3	71.9	0.028	8.73 (2.41, 31.59)	0.001	0.137
Chronic HBV	4	41.1	0.165	3.06 (1.11, 8.46)	0.031	0.370
